# “OUR ELDERS LOVE WHEN WE MAKE THE BUFFALO DINNERS AND SOUP”: TRADITIONAL FOODS AND TITLE VI NUTRITION PROGRAMS

**DOI:** 10.1093/geroni/igae098.3446

**Published:** 2024-12-31

**Authors:** Traci Wilson

**Affiliations:** USAging, Washington, District of Columbia, United States

## Abstract

Title VI Native American Aging Programs (Title VI programs), funded by Title VI of the Older Americans Act, support the health, wellness and social engagement needs of Native elders by providing nutrition, supportive services and caregiver supports to American Indian, Alaska Native and Native Hawaiian people. There are over 290 Title VI grantees, representing a diverse group of more than 400 tribal nations. Title VI programs provide culturally-relevant services to address the health-related social needs of elders, such as meals, transportation, in-home services, social engagement opportunities, and family caregiver supports, helping to ensure that elders can continue to live in their homes and communities as long as possible. A large portion of Title VI funding is allocated to nutrition programs, including congregate and home-delivered meals. Every three years, USAging, with funding from the Administration for Community Living, surveys Title VI directors about their program funding, staffing, services, and unmet needs. The most recent survey, conducted in late 2023 with an 80% response rate, included a new module on if and how Title VI nutrition programs incorporate traditional foods. This poster will describe the prevalence and types of traditional foods in OAA Title VI nutrition programs, involvement of elders in providing recipes and passing on knowledge, and the challenges and successes Title VI programs have when sourcing and incorporating traditional foods into their nutrition programs.

